# Deepening the understanding of CNVs on chromosome 15q11–13 by using hiPSCs: An overview

**DOI:** 10.3389/fcell.2022.1107881

**Published:** 2023-01-06

**Authors:** Angela Maria Giada Giovenale, Giorgia Ruotolo, Amata Amy Soriano, Elisa Maria Turco, Giovannina Rotundo, Alessia Casamassa, Angela D’Anzi, Angelo Luigi Vescovi, Jessica Rosati

**Affiliations:** ^1^ Cellular Reprogramming Unit, Fondazione IRCCS Casa Sollievo della Sofferenza, San Giovanni Rotondo, Italy; ^2^ Department of Biotechnology and Biosciences, University of Milano-Bicocca, Milan, Italy

**Keywords:** neurodevelopmental disorders, neuropsychiatric disorders, 15q11-13, CHRNA7, nicotinic acetylcholine receptor, copy number variation, CNV

## Abstract

The human α7 neuronal nicotinic acetylcholine receptor gene (CHRNA7) is widely expressed in the central and peripheral nervous systems. This receptor is implicated in both brain development and adult neurogenesis thanks to its ability to mediate acetylcholine stimulus (Ach). Copy number variations (CNVs) of CHRNA7 gene have been identified in humans and are genetically linked to cognitive impairments associated with multiple disorders, including schizophrenia, bipolar disorder, epilepsy, Alzheimer’s disease, and others. Currently, α7 receptor analysis has been commonly performed in animal models due to the impossibility of direct investigation of the living human brain. But the use of model systems has shown that there are very large differences between humans and mice when researchers must study the CNVs and, in particular, the CNV of chromosome 15q13.3 where the CHRNA7 gene is present. In fact, human beings present genomic alterations as well as the presence of genes of recent origin that are not present in other model systems as well as they show a very heterogeneous symptomatology that is associated with both their genetic background and the environment where they live. To date, the induced pluripotent stem cells, obtained from patients carrying CNV in CHRNA7 gene, are a good *in vitro* model for studying the association of the α7 receptor to human diseases. In this review, we will outline the current state of hiPSCs technology applications in neurological diseases caused by CNVs in CHRNA7 gene. Furthermore, we will discuss some weaknesses that emerge from the overall analysis of the published articles.

## Introduction

The major contribution to the variability of the human genome comes from DNA deletions and duplications larger than 1 Kb of one or both alleles, defined as copy number variations (CNVs) (Zarrei et al., 2015). These quantitative structural variants can be located in gene-rich or poor chromosome regions (Redon et al., 2006). CNVs can be grouped into three main categories of clinical relevance (Nowakowska, 2017): 1) Benign variants that do not have a negative effect on human phenotype (Tuzun et al., 2005), (Sharp et al., 2005) and constitute an important source for human phenotypic variability (Pinto et al., 2007). 2) Pathogenic variants that are associated with a wide spectrum of diseases. CNVs can change human phenotypes by acting through various rearrangements of genome structure that include: duplications or deletions of dosage-sensitive genes; rearranged breakpoints that interrupt and inactivate genes; generation of fusion genes at the breakpoints with a gain-of-function; unmasking of recessive mutations or of transvections, when CNV, occurring in one chromosome, affects an allele on the other chromosome at the same locus. CNVs are pathological even when they occur in non-coding regions (“position effect” variation) involved in gene expression regulation (Lupski and Stankiewicz, 2005). 3) Variants of uncertain significance (VOUS), with an ambiguous clinical relevance that does not permit classification as pathogenic or benign (Vermeesch et al., 2012).

Many of these quantitative structural variants happen in correspondence with chromosome 15q11–13, which shows a high frequency of genomic rearrangements due to its intrinsic genomic instability (Makoff and Flomen, 2007; Rosenfeld et al., 2011). The proximal region of the long arm of chromosome 15 is characterized by three segmental duplications that are subject to Non-Allelic Homologous Recombination (NAHR), due to their high homology. These chromosome regions, for their characteristics, are known as BreakPoint regions (BP3, BP4, BP5) since, after NAHR, they determine chromosomal rearrangements, such as microdeletions and microduplications. Rearrangements of chromosome 15q11-13 are associated with Prader-Willi and Angelman syndromes and neurological disorders (Makoff and Flomen, 2007; Sharp et al., 2008; Miller et al., 2009; Rosenfeld et al., 2011). Generally, CNVs affecting 15q13.3 have been reported correlated to a highly variable phenotype and different neurological manifestations (Budisteanu et al., 2021). Among these, both chromosome 15q13.3 deletions and duplications are associated with autism spectrum disorder (ASD), intellectual disability (ID)/developmental delay (DD), mood disorders, speech problems, and schizophrenia (SCZ) (Sharp et al., 2008; Miller et al., 2009; van Bon et al., 2009; Szafranski et al., 2010; Beal, 2014; Szatkiewicz et al., 2014; Bacchelli et al., 2015; Lowther et al., 2015; Pettigrew et al., 2015; Zhou et al., 2016). On the one hand, CNVs involving deletion generally exhibit severe, highly penetrant patient phenotypes; for instance, 15q13.3 microdeletions are usually associated with cognitive deficits, behavioral abnormalities, and ASD (Ben-Shachar et al., 2009; Miller et al., 2009; Pagnamenta et al., 2009; van Bon et al., 2009; Masurel-Paulet et al., 2010). On the other hand, CNVs involving duplication often cause widely variable and less penetrant phenotypic expressivity among affected subjects (Mefford et al., 2012; Newman et al., 2015). 15q13.3 microduplications are indeed correlated to milder clinical phenotypes, including borderline ID, ASD and attention deficit hyperactivity disorder (ADHD) (Williams et al., 2012; Gillentine and Schaaf, 2015; Meganathan et al., 2021). However, 15q13.3 microduplications are frequent both in clinical and non-clinical cases (symptomatic and non-symptomatic), making their contribution to pathogenicity hard to estimate (Wiśniowiecka-Kowalnik et al., 2013; Coe et al., 2014; Rehm et al., 2015; Gillentine et al., 2017). This region is important because it is here that deletions or duplications affect the human α7 nicotinic cholinergic receptor (*CHRNA7*) gene. The situation is further complicated by the presence of the chimeric fusion gene *CHRFAM7A*, specific only to human beings, responsible for variations in nAChR structure that impacts patient phenotypes in a variable way (Araud et al., 2011). Consistent with new evidence suggesting that the expression of CHRFAM7A fusion gene dominant-negatively inhibits the channel functions of α7-nAChR, accumulating data have shown an association between *CHRFAM7A* dosage and Alzheimer’s disease, nicotine dependence, schizophrenia, bipolar disorder, and other neuropsychiatric disorders (Ihnatovych et al., 2019; Peng et al., 2022).

Multiple animal model systems have been used to study the impact of the 15q13.3 CNVs on the human brain, but they have failed to fully recapitulate human phenotypes, perhaps due to the different genes present in this chromosome region. Thus, induced pluripotent stem cells (iPSCs) provide a useful human model to understand the pathological mechanisms involving these CNVs (Gillentine, 2022). This review will be focused both on the use of hiPSCs technology for the comprehension of 15q13.3 CNVs-dependent neurological diseases and on future applications of iPSCs in drug development for the abovementioned disorders and diseases.

### A genomic and structural overview of the α7 nicotinic cholinergic receptor

The human (*CHRNA7*) gene includes ten exons and encodes for the α7 subunit of neuronal nicotinic Acetylcholine Receptor (α7nAChR), widely expressed both in central and peripheral nervous systems. On the cell surface, five α7nAChR monomers associate to form an oligomeric ligand-gated ion channel, belonging to the Nicotinic Acetylcholine Receptor (nAChRs) family. nAChRs are selective transmembrane ion channels for Na+, K+, and Ca2+, which bind neurotransmitters and regulate excitatory and inhibitory signaling. They can be composed of α or β subunits, creating both homo- and hetero-pentameric channels. In particular, α7nAChR is characterized by a low affinity for nicotine, a high affinity for α-bungarotoxin (α -Bgtx), high permeability to calcium (Marks and Collins, 1982), and fast desensitization (Zhang et al., 2011). The α7 receptor is expressed in presynaptic neurons and astrocytes (Vijayaraghavan et al., 1992; Patel et al., 2017) where it modulates neurotransmitter release, as well as in post-synaptic regions, influencing gene expression. Based on these functions, α7nAChR is implicated in cognitive processes and synaptic plasticity (Shen and Yakel, 2009), neurotransmitter release and immune responsiveness with consequent implications for Alzheimer’s disease, Parkinson’s disease and so on (Schaaf, 2014; Quik et al., 2015; Liu et al., 2017). Moreover, its expression in extra-neuronal tissues indicates an additional role in modulating several calcium-activated signal pathways influencing proliferation, differentiation, migration, and inflammatory response (Wang et al., 2003; Campbell et al., 2010; Mucchietto et al., 2018).

**FIGURE 1:**
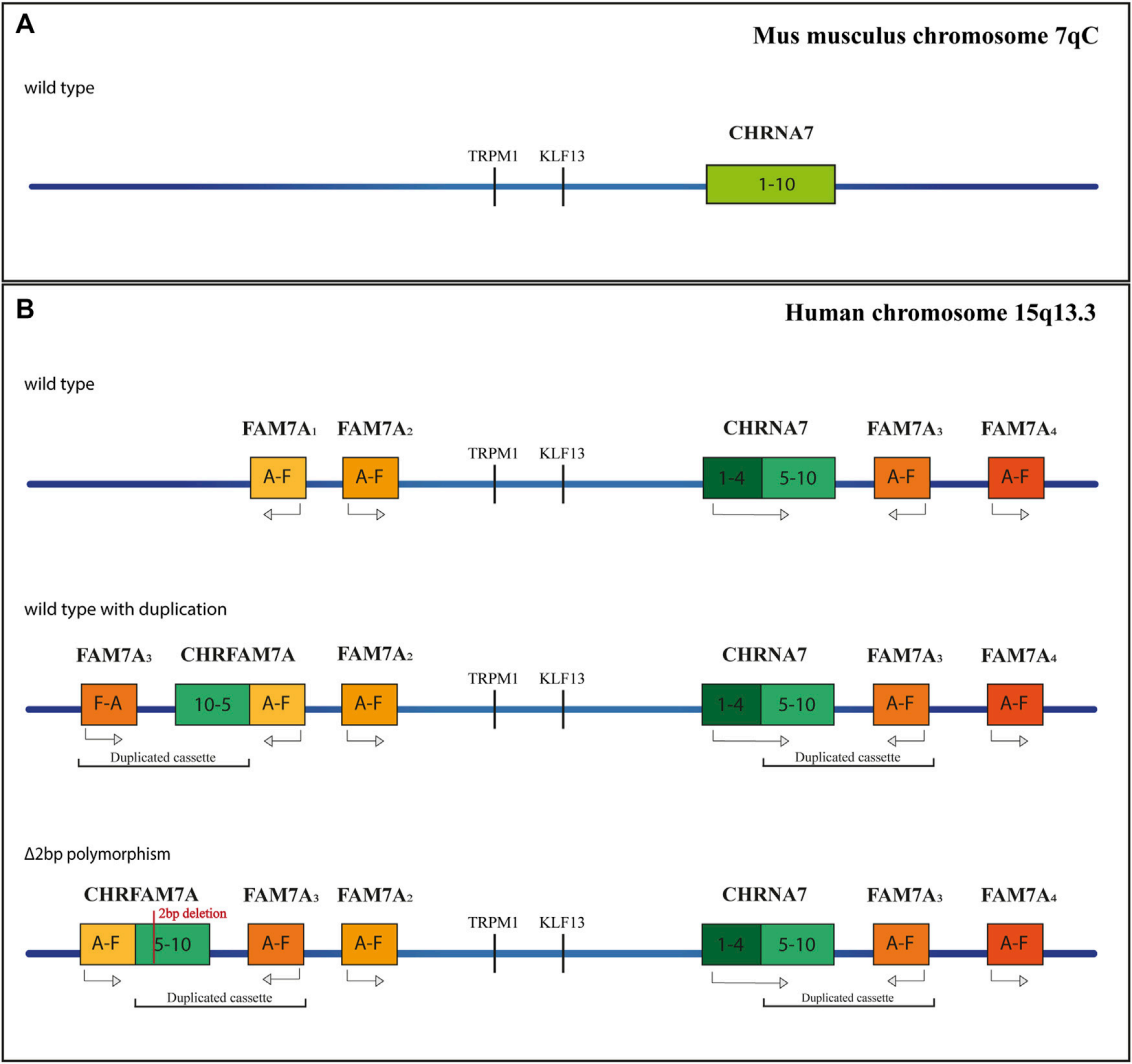
Schematic representation of the CHRNA7 locus. **(A)** Orthologus locus in mus musculus, at the 7qC chromosome. **(B)** Different genetic profiles of the human Chromosome 15q13.3, without duplication, with the chimeric gene and with the 2bp mutation.

A second distinct α7nAChR gene was discovered in the human genome in 1998 (Gault et al., 1998). Close to *CHRNA7*, there are four repeated sequences, called *FAM7A*, made up of exons A, B, C, and E, copies of two exons of the Unc-51 Like Kinase 4 gene (*ULK4*) (Lang et al., 2014), and exons D and F, homologous to the *GOLGA8B* gene (Stephens et al., 2012). The duplication of exons 5–10 of *CHRNA7* together with a *FAM7A* sequence, and the subsequent insertion of this cassette at 1.6 Mb upstream of *CHRNA7*, provoke the formation of the chimeric gene *CHRFAM7A* (Figure 1). The duplicated cassette has an inverted orientation concerning the *CHRNA7* gene; furthermore, a 2bp deletion polymorphism is present, in which the *CHRFAM7AΔ2bp* insertion also results in an inverted orientation (Flomen et al., 2008). This new chimeric gene is largely expressed in both CNS and peripheral tissues and is present in about 90%–95% of individuals, 30% of whom have a single copy instead of two alleles (Riley et al., 2002; Sinkus et al., 2009). Homologous *CHRNA7* has not been found in primates and rodents (Locke et al., 2003), indicating recent origins. *CHRFAM7A* presents an Open Reading Frame (ORF) in correspondence with the exon B, and the translation product results in a smaller protein than the original *CHRNA7* (46 kDa instead of 57 kDa), lacking part of the N-terminal domain that contains the signal peptide and part of the agonist-binding site. A second isoform originates from the ORF in exon 6 and produces a shorter peptide (38 kDa), lacking the ligand peptide and the entire binding site. The transcription of *CHRFAM7A* does not affect the expression of *CHRNA7*, and even though *CHRFAM7A* has a lower transcription rate in the brain, the encoded protein (dupα7) has a dominant negative effect on the α7 subunits (de Lucas-Cerrillo et al., 2011; Chan et al., 2019). There are two possible explanations for this phenotype. The first is that the dupα7 protein preferentially localizes at the endoplasmic reticulum, where it might sequester the α7 subunits and reduce the α7nAChR activity (Araud et al., 2011). The second mechanism might involve the assembly of dupα7 with other α7 subunits in the cellular membrane. When homomeric, Dupα7 receptors are not functional, as ligands cannot bind to them, but *in vitro* experiments have shown the formation of heteromeric ion channels (Wang et al., 2014). The efficiency of receptors is guaranteed as long as there are three dupα7 adjacent to α7 subunits, which allow the formation of dupα7/α7 interfaces, containing the agonist binding site. The presence of *CHRFAM7A* in double copy, or the presence of the 2bp depleted polymorphism could influence the overall function of α7 receptors.

### Animal models currently in use

Multiple animal model systems have been used to determine the role and function of CHRNA7 in human phenotypes. The first studies to explore putative regulatory mechanisms of α7nAChR (de Lucas-Cerrillo et al., 2011) were carried out on *Xenopus laevis* oocytes and rat cell lines previously transfected with two constructs carrying *CHRFAM7A* and *CHRFAM7AΔ2bp* (de Lucas-Cerrillo et al., 2011). These experiments highlighted that the chimeric gene *CHRFAM7A* negatively regulates α7nAChR functions*,* especially in the presence of partial duplication with the 2-bp deletion, *CHRFAM7AΔ2bp* (Araud et al., 2011). Currently, murine models are mainly used for the 15q13.3 CNV study (Fejgin et al., 2014; Lewis et al., 2018; Felix et al., 2019). The first *Chrna7-*deficient mice showed attention deficits, anxiety, and anhedonia (Zhang et al., 2016) but this model was found to be unsuitable for recapitulating human pathological phenotypes associated with learning, memory, and sensorimotor gating (Yin et al., 2017). Further studies on *Chrna7* knockout mice highlighted altered temporal processing of the auditory brainstem response (ABR) signal that may contribute to degraded spike timing in the midbrain (Felix et al., 2019). However, experiments on *Chrna7* knockout mice did not provide significant information owing to the several essential differences between humans and murine species: first, behavioral and functional defects were observed in homozygous knockout mice, while in humans, the 15q13.3 CNV are generally heterozygous; second, the Endoplasmic Reticulum (ER) chaperone RIC3 involved in nAChRs assembly in human beings is expressed differently than in mice (Halevi et al., 2003), suggesting a probable functional difference in α7nAChR assembly; third, the negative modulator *CHRFAM7A* is only expressed in humans, indicating that different regulatory mechanisms are probably involved in mice (Yin et al., 2017). Preliminary studies on *CHRFAM7A* transgenic mice have highlighted a different expression of proteins involved in the signaling pathways at the basis of PD, AD, Huntington’s disease (HD), and alcoholism such as calcium signaling, oxidative phosphorylation and others. It suggests that the *CHRFAM7A* gene contributes to the pathological process likely through precise control of α7-nAChR functions in the brain (Yin et al., 2017; Jiang et al., 2019). However, the role of CHRFAM7A in 15q.13.3 CNV needs to be explored more in depth, using a human cellular model such as iPSCs.

### Deepening understanding with the hiPSC model

**FIGURE 2:**
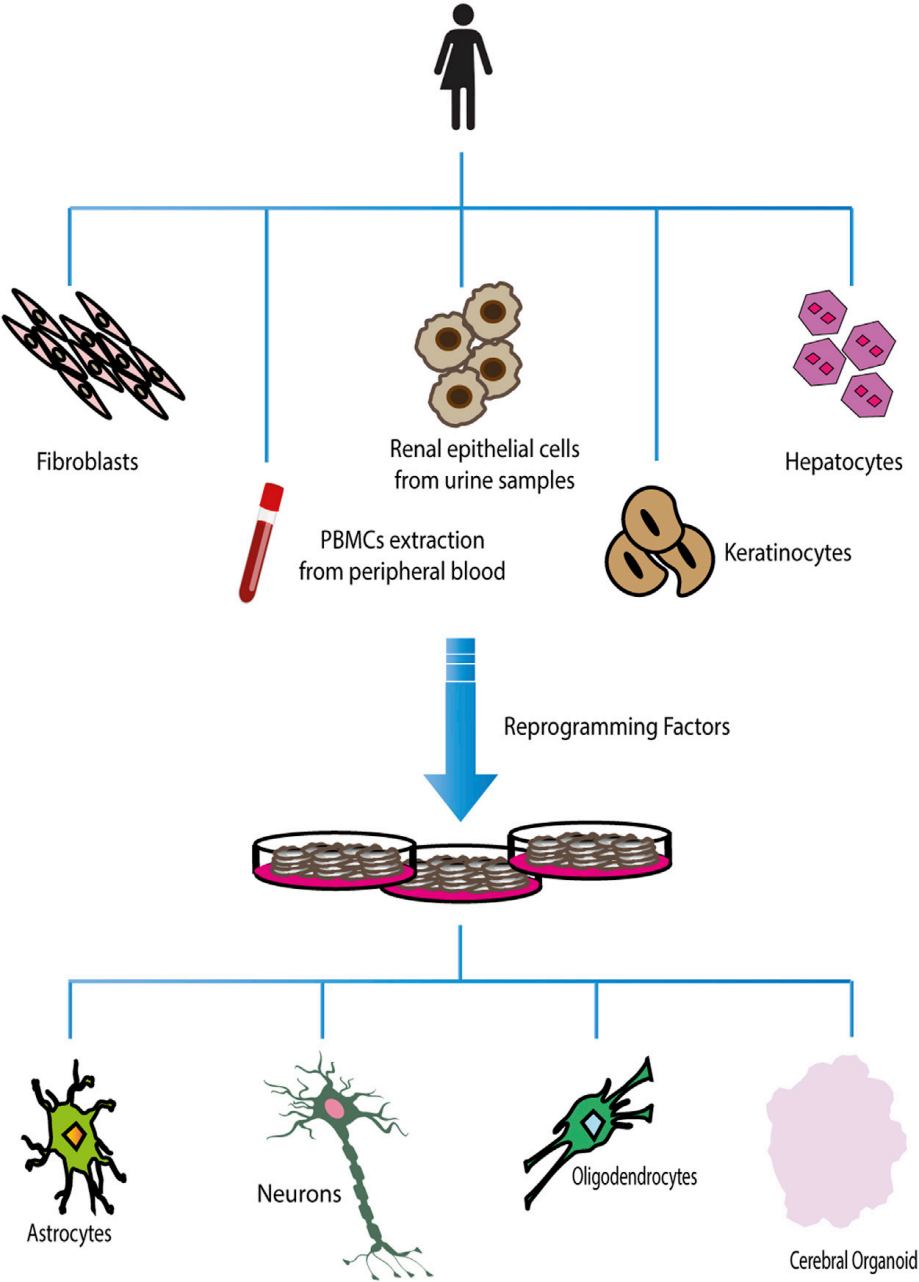
Reprogramming and differentiation of hiPSCs. Different adult somatic cells can be reprogrammed in induced Pluripotent Stem Cells. Once hiPSCs have been obtained, they can be used to generate all types of terminally differentiated cells.

The advent of cellular reprogramming technology has proven that induced Pluripotent Stem Cells (iPSCs) can represent an extremely useful resource for studying neurodevelopmental/neuropsychiatric diseases. Human-induced Pluripotent Stem Cells (hiPSCs) are generated by reprogramming somatic cells into pluripotent stem cells, which can be further differentiated into a large variety of cell types, such as neurons, astrocytes, and oligodendrocytes (Dimos et al., 2008; Chambers et al., 2009) (Figure 2). To date, it is possible to reprogram a large number of cell types, such as fibroblasts (skin biopsies), keratinocytes (hair roots) (Piao et al., 2014; Soliman et al., 2017), cord blood endothelial cells (Haase et al., 2009), blood cells (Ye et al., 2009), T lymphocytes (Brown et al., 2010; Seki et al., 2011), melanocytes (Utikal et al., 2009), hepatocytes (Liu et al., 2010), mesangial cells (Song et al., 2011), exfoliated renal epithelial cells from urine samples (Ruiz et al., 2010; Zhou et al., 2012). A growing number of studies have demonstrated that iPSCs are an excellent cellular model for studying both syndromic and idiopathic forms of neurodevelopmental/neuropsychiatric disorders, which have their origin in the prenatal period during cell differentiation (Soliman et al., 2017). Furthermore, iPSC-derived neurons can be generated from patients carrying a specific genetic background, corresponding to a particular neuropsychiatric disease, where rare but penetrant genetic abnormalities are likely to play a role (Park et al., 2008; Marchetto et al., 2010; Dolmetsch and Geschwind, 2011; Grskovic et al., 2011; Paşca et al., 2011). Recently, the production of iPSC-derived 3D organoids has allowed researchers to investigate the interaction of multiple cell types in a more brain-like microenvironment. This model takes advantage of the capability of hiPSCs to self-organize into embryoid bodies and subsequently differentiate into organoids in response to environmental cues mimicking *in vivo* conditions. The use of human neural organoids allows researchers to study, *in vitro*, both the three-dimensional (3D) cytoarchitecture of the human brain and its development, overcoming the limitations associated with the use of animal models (Guy et al., 2021). To date, many neurodevelopmental and neurodegenerative disorders such as primary microcephaly (Lancaster et al., 2013; Li et al., 2017a; Zhang et al., 2019), macrocephaly (Li et al., 2017b), autism spectrum disorder (Mariani et al., 2015; Schafer et al., 2019), Parkinson’s disease (PD) (Kim et al., 2019; Smits et al., 2019), and Alzheimer’s disease (Raja et al., 2016; Gonzalez et al., 2018; Lin et al., 2018; Ghatak et al., 2019) were studied using this technology.

### Influence of the genomic background on the physiological behavior of α7nAChR

To date, very few studies have been published regarding the expression and functionality of nAChRs in human iPSC-derived neurons under physiological conditions. Based on a previous study (Gill et al., 2013), in 2015 researchers profiled the electrophysiological properties of GABA neurons (catalog number R1013, Cellular Dynamics International) (Chatzidaki et al., 2015). In these iPSC-derived cells, they confirmed the expression of both *CHRNA7* and *CHRFAM7A* genes, demonstrating that hiPSC-derived neurons recapitulated the main features of the α7nAChR: low open probability (the tendency for fewer receptors to open) and fast desensitization, characteristics that could be inverted with the addition of positive allosteric modulators (PAM type II) together with selective agonists, as observed in HEK 293 cells (DaCosta et al., 2011), *Xenopus laevis* Oocytes (Pałczyńska et al., 2012) and BOSC 23 Cells (Williams et al., 2011). In the second study (Larsen et al., 2019), three different clones belonging to the same reprogramming event (BIONi010-A, BIONi010-B, and BIONi010-C, European Bank of induced Pluripotent Stem Cells) were obtained from fibroblasts of a healthy subject. They observed an increase in the expression of *CHRNA7* and *CHRFAM7A* during neuronal maturation, highlighting the player role of these genes during neural differentiation. The interesting outcome of this work was the formation of a neuronal network model that offered the possibility of evaluating α7 receptor involvement during synaptic transmission. These papers demonstrated that hiPSC-derived neurons not only highlight features of α7nAChR commonly observed in other models but represent a more specific representation of human conditions that could evidence and clarify new mechanisms not already well characterized. Nevertheless, the important outcome of these two works is that even using different reprogramming and differentiation protocols, both models recapitulate the same receptor features, confirming the reproducibility of this model.

Since the regulation of α7nAChR is complicated by the presence of the *CHRFAM7A* fusion gene in humans, understanding its influence on α7nAChR function is useful for preclinical studies. This motivation prompted Ihnatovych and collaborators in 2019 to undertake an interesting study in which they evaluated how the amount of CHRFAM7A influences the receptor functions (Ihnatovych et al., 2019). Fibroblasts were reprogrammed from two subjects affected by Alzheimer’s Disease (AD), carrying respectively 0 (ancestral haplotype) and 1 copy of the *CHRFAM7A* gene (UB068-0 copy, UB052-1 copy). These hiPSCs were differentiated into medial ganglionic eminence (MGE) progenitors and neurons. They found a stable *CHRNA7* expression during differentiation over 40 days in the 0-copy line and overexpression of both *CHRNA7* and *CHRFAM7A* mRNAs in the 1-copy cell line, perhaps as possible compensation for the negative modulation of the chimeric gene. Moreover, the presence of CHRFAM7A modified the kinetics of channel opening in pharmacological assays with PNU 120596, showing faster desensitization of the channel in 1-copy cells than in 0-copy cells. As Alzheimer’s patients accumulate amyloid beta (Aβ), which CHRNA7 binds to, and the researchers observed a decrease in the uptake of the Aβ1-42 peptide in the 1-copy line in a dosage-dependent manner, they concluded this could be a possible protective effect under physiological conditions.

The group subsequently published two other papers (Ihnatovych et al., 2020; Szigeti et al., 2020), exploring electrophysiology and Aβ uptake in the first study and the anti-inflammatory effects of microglia (MGL) in the second, in the presence and absence of the *CHRFAM7A* allele and presence and absence of *CHRFAM7Abp*. The Aβ uptake was quantified by transfecting MGE progenitors with an empty vector (EV), CHRFAM7A, and CHRFAM7Abp cDNAs. CHRFAM7A transfected cells showed a mitigated Aβ_1-42_ uptake compared to EV and *CHRFAM7Abp* transfected cells, suggesting that while *CHRFAM7A* is a dominant negative modulator, the inverted *CHRFAM7Abp* has the same functions of a null allele for the two AD relevant phenotypes analyzed. UB068 (*CHRFAM7A* null) hiPSCs were then genome-edited and differentiated into MGE progenitors. The comparison between UB068 (*CHRFAM7A* null) and the two *CHRFAM7A* carrier lines, UB052 and UB068_CHRFAM7A, demonstrated different responses to Acetylcholine Esterase Inhibitors (AChEIs) and encenicline exposure. In particular, the Aβ_1-42_ uptake was linear with a dose of AChEIs and decreased in presence of encenicline only in UB068. These data were very interesting because they highlighted how only carriers of ancestral haplotype could benefit from α7nAChR targeting therapies. The information obtained from patients’ iPSC-derived cells is highly transferable to humans: in fact, starting from a genotyping of 1,174 subjects that split the population 1:3 for non-carriers to carriers of the direct *CHRFAM7A* functional allele, Ihnatovych and his group studied the response to initiation of AChEI therapy and delayed disease modifying treatment (DMT) effect over a 6-year observation period. The analysis confirmed preliminary pharmacological data on cells, showing that carriers with 0 copies of the CHRFAM7A had both a superior response to first exposure and a greater DMT effect from AChEI. The complex study conducted by the research group over the years has bridged the gap in cholinergic strategies in AD, showing that not considering *CHRFAM7A* could lead to the rejection of drug candidates that could be beneficial for 25% of AD patients (Szigeti et al., 2020).

In the second paper, the authors analyzed the internalization of fluorescent-Aβ in all microglial cell lines (MGL) finding that cells with *CHRFAM7A* presented a reduction of the dose-response curve of Aβ internalization. They also treated MGL with mecamylamine (MLA, a nicotinic acetylcholine receptor antagonist) and evaluated their internalization activity of the Aβ peptide: despite an increase of internalization of Aβ in the 0-copy line, no differences were detected for lines carrying *CHRFAM7A*. This suggested that MLA had a pro-inflammatory effect through the activation of Microglia cells. The activation of MGL cells leads to the translocation of p65 (representing the NF-kB activation pathway) in nuclei and consequently the expression of pro-inflammatory genes. Cells carrying *CHRFAM7A* showed an increase of p65 with respect to the line without fusion gene; however, the treatment with MLA caused an increase of p65 only in the 0-copy line concerning basal condition. This suggested that CHRNA7 had an anti-inflammatory action while CHRFAM7A possessed a pro-inflammatory activity, as a consequence of its negative effect on CHRNA7 (Ihnatovych et al., 2020). To completely clarify the molecular mechanism of the effects of *CHRFAM7A* it would have been appropriate to include a control line carrying a double copy of the fusion gene, which represents the major polymorphism in the population.

### Impact of CNVs (duplications/deletions) on the function of α7nAChR

**FIGURE 3:**
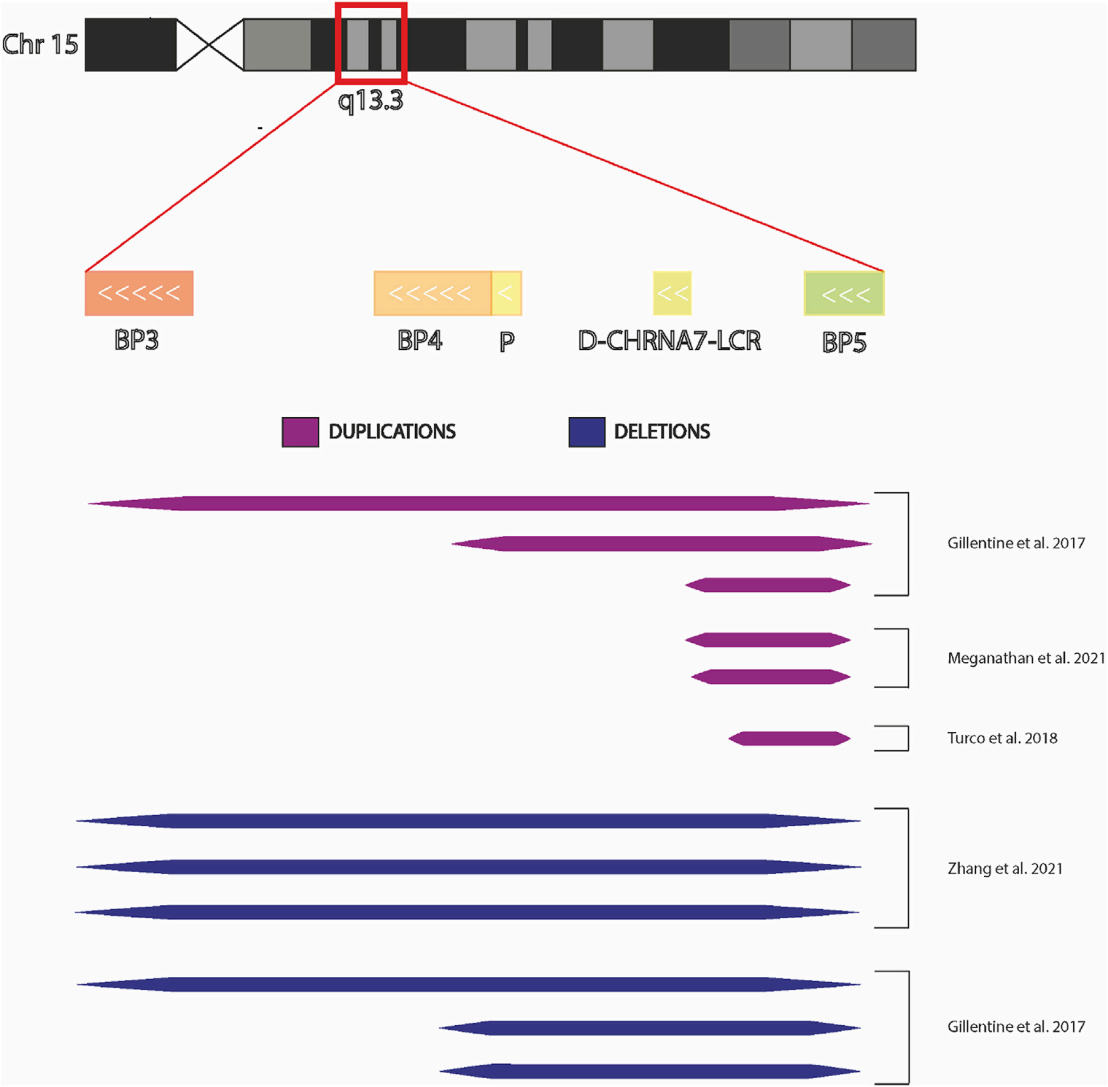
Chromosome 15q13.3 and hiPSCs derived from individuals with CNV. Graphic representation of chromosomal region 15q13.3 showing BreakPoint regions BP3, BP4, and BP5 and the extensions the microdeletions and microduplications present in the hiPSCs in the published studies.

Patient-derived induced Pluripotent Stem Cells (iPSCs) technology can be a potentially limitless useful source of human cells for investigating molecular mechanisms underlying neurological diseases. To better evaluate the potential implication of CHRNA7 and CHRFAM7A dosages in such processes, here we summarized a state of the art of human iPSCs and iPSC-derived models from subjects carrying CNVs at 15q13.3 (Figure 3).

In 2017, Gillentine and collaborators used somatic cells belonging to probands carrying 15q13.3 CNV for the first time to produce induced pluripotent stem cells to differentiate into Neural Progenitors Cells (NPCs) (Gillentine et al., 2017). These cortical-like NPCs were utilized to investigate the molecular consequences of *CHRNA7* copy-number variation, overcoming the limitations of animal models. The subjects who participated in the study were three individuals with 15q13.3 deletions (two of which spanned BP4/BP5 and one spanned BP3/BP5), three individuals with 15q13.3 duplications (the first spanned BP4/BP5, the second spanned BP3/BP5 and the last duplication spanned +9 D-CHRNA7-LCR/BP5) and three copy neutral control lines. All selected patients had similar ages and phenotypic variability ranging from ID/DD, ASD to ADHD; only the last duplication (the shorter one) resulted asymptomatic.

The authors explored gene expression of *CHRNA7*, α7 nAChR-dependent calcium flux, and the consequences of altered calcium signaling. As expected, the qPCR analysis showed a significant decrease of about 50% in *CHRNA7* expression compared to controls both in hiPSCs and NPCs carrying the deletions. To characterize the duplications, they used primers specific for the duplicated portion of *CHRNA7* in the asymptomatic proband and identified an increased expression of *CHRNA7* in all three lines compared to the controls.

The great potentiality of the use of human iPSCs was demonstrated through the FLIPR Tetra High-Throughput Cellular Screening System (Molecular Devices) with which the authors co-treated the NPCs with two drugs, 1 μM epibatidine and 3 μM PNU-120596 (a positive allosteric modulator), to accurately detect α7 nAChR-specific calcium flux. The analysis showed a decreased calcium flux in both groups of NPCs; a reduction was also confirmed from the downregulation of downstream effectors, such as the JAK2-PI3K pathway known for its role in modulating neuronal excitability and neurotransmitter release. Although this result was expected in the probands carrying the deletion, it was not expected in the lines with duplication. From the observation of general deregulation of all other nAChRs in these cells, the authors decided to verify two possible hypotheses. Having observed that calcium flux did not vary among CNV and controls, despite general deregulation of all other nAChRs in these cells, the authors hypothesized an altered function of the chaperons *RIC3* and *NACHO* necessary to the nAChR receptors assembly, trafficking, and cell surface expression. Gene expression analysis demonstrated an upregulation of both mRNAs in duplicated NPCs samples while there was a downregulation in deleted NPCs samples. Since the altered expression of these two nAChRs-specific chaperons is related to the stress of Endoplasmic Reticulum (ER) (Severance and Yolken, 2007; Srinivasan et al., 2012; Lewis and Picciotto, 2013), the authors explored two pathways, PERK activation and IREα splicing of XBP1, associated with ER stress. In particular, the levels of the spliced form of XBP1 resulted increased in NPCs with duplications, but similar to controls in NPCs with deletions. The reduced expression of *CHRNA7* on the cell surface and the consequent calcium flux alteration found in NPC with duplication was therefore due to the accumulation of proteins in the endoplasmic reticulum. The authors had unexpectedly found two different mechanisms with the same result, in cells carrying deletion and duplication of the 15q13.3 region.

In 2018 another study was published by Turco et al. on an iPSC line carrying a 15q13.3 duplication. Genotype analysis showed that the current duplication involved only the *CHRNA7* gene. This evidence, in addition to the healthy phenotype of the subject, suggests that duplications of the single gene are not sufficient for the onset of the phenotype, by what was already observed in the asymptomatic proband (Gillentine et al., 2017). Further studies carried out on this apparently healthy duplication will be useful to clarify the mechanisms at the base of pathologic phenotypes.

Variable penetrance could explain why individuals carrying the same duplication from a partially shared genetic background present different phenotypes. In order to understand this phenomenon, Meganathan and collaborators selected a family of four individuals, including three subjects with the same 15q13.3 duplication: the mother, who had no clinical diagnosis (UM), her older son, who exhibited distinct features of autism and emotional dysregulation (the affected proband, AP), and her younger affected son, who exhibited mild ASD, ADHD, and mood disorder traits, while the father did not carry the duplication. The CNV in these three subjects was a duplication of −400 kilobase at chromosome 15, band q13.3; the only gene located in the duplicated region was *CHRNA7*. Three clonal hiPSC lines per subject (from UM and AP) were produced, while single clonal hiPSC lines derived from unrelated, unaffected male and female donors (UC-M and UC-F) were used as control subjects. hiPSCs were differentiated into cortical Excitatory Neurons (cExN), cortical Inhibitory interNeurons (cIN), and neural progenitor cells (cExNPCs and cINPCs), then combined at a 1:1 ratio to generate cortical neural organoids.

The authors observed disruptions of the correct cellular physiology and reduced neurodevelopment in the AP-derived model, while it was not observed in either the UM or unrelated healthy controls. Neuronal gene expression was dysregulated in the AP, including reduced expression of genes related to behavior, psychological disorders, neuritogenesis, neuronal migration, WNT pathway, axonal guidance, and GABA receptor signaling. This dysregulation influenced cellular functioning, resulting in increased neural progenitor proliferation, impaired neuronal differentiation, maturation, and migration, and increased endoplasmic reticulum (ER) stress. Both the neuronal migration deficit and elevated ER stress were selectively rescued by different pharmacologic agents. The UM model instead exhibited upregulated expression of genes in many of the same pathways, suggesting that molecular compensation could have contributed to the lack of neurodevelopmental phenotypes in this model. However, both AP- and UM-derived neurons exhibited shared alterations of neuronal function, including increased action-potential firing and elevated cholinergic activity, consistent with increased homomeric CHRNA7 channel activity (Meganathan et al., 2021).

This research presents certain limitations: in particular, despite the genotyping analysis, the expression of the CHRNA7 gene, and also of the chimeric gene, was not observed. Moreover, the electrophysiological analysis only evaluates an alteration in the flux activity when induced by Ach and choline, without using different allosteric modulators, which are essential for confirming that calcium influx depends on the α7 Receptor, excluding the participation of other nAChR members. The authors did not exclude two possible hypotheses: whether the observed alterations were directly associated with alterations of CHRNA7 activity or due to indirect mechanisms that might have affected the functionality of different nACh Receptors. Further studies will be necessary to clarify the role of CHRNA7 in these pathological phenotypes.

In 2021, Zhang and colleagues reprogrammed six fibroblast lines into hiPSC (three of the lines belonged to individuals with 15q13.3 microdeletions and the others belonged to healthy individuals), to study the effects of the 15q13.3 microdeletion on genome-wide gene expression, DNA methylation, chromatin accessibility, and sensitivity to cisplatin-induced DNA damage. In all three patients, the heterozygous 15q13.3 microdeletions were detected between breakpoints 3 and 4 (BP3/BP4). The iPSCs were differentiated into induced Neurons (iNs) using the neurogenin-2 induction method. To determine the effects of 15q13.3 microdeletions on gene expression of Chr15, they performed RNA-seq on iPSCs and iNs of 6 cell lines. Using Genome-wide analyses, they found 178 Differentially Expressed Genes (DEGs) in hiPSCs and 369 in iNs with 15q13.3 microdeletions. Some of the DEGs found in iNs are known to be involved in neuropsychiatric disorders, such as: CACNG3, SCN8A, SPATA5, and for intellectual disability. No enriched Gene Ontology (GO) terms were found in hiPSCs, while in iNs 46 significant Biological Process (GOBP) and 7 Molecular Function (GOMF) terms were identified. These enriched GO terms belong to different processes related to a neurodevelopmental disease such as WNT binding, ribosome biogenesis, and DNA repair and DNA binding. The researchers focused their attention on the DNA repair pathway, evaluating the cell survival of iNs in response to DNA damage induced by cisplatin. Cells carrying the 15q13.3 microdeletion showed a reduced survival rate compared with control iNs, demonstrating a high susceptibility to DNA damage, as previously observed in cortex derived from the murine model (Gordon et al., 2021). In addition, the authors identified: 1) A differential methylation state of protocadherins, which, modulating the WNT pathway, are implicated in various neurodevelopmental processes, including synapse assembly, neuronal differentiation, and neurotransmission. 2) reduced accessibility in the regulatory binding sites of the genes implicated in various neuropsychiatric disorders. 3) The consequences of the 15q13.3 microdeletion were not associated with the haploinsufficiency of a single gene but with the combination of various genes located in the CNV; this observation was possible using CRISPR/cas9 technique to generate cell lines carrying a homologous deletion of every single gene presents in the CNV. This could explain the variability of pathological phenotypes observed in individuals with different 15q13.3 CNVs (Zhang et al., 2021). Nevertheless, in order to correlate the pathological phenotype with the CNV and not with the patient’s background, Zhang and colleagues should have created the specific microdeletions by the CRISP/cas9 technique in iPSCs derived from healthy donors (Tai et al., 2016).

**FIGURE 4:**
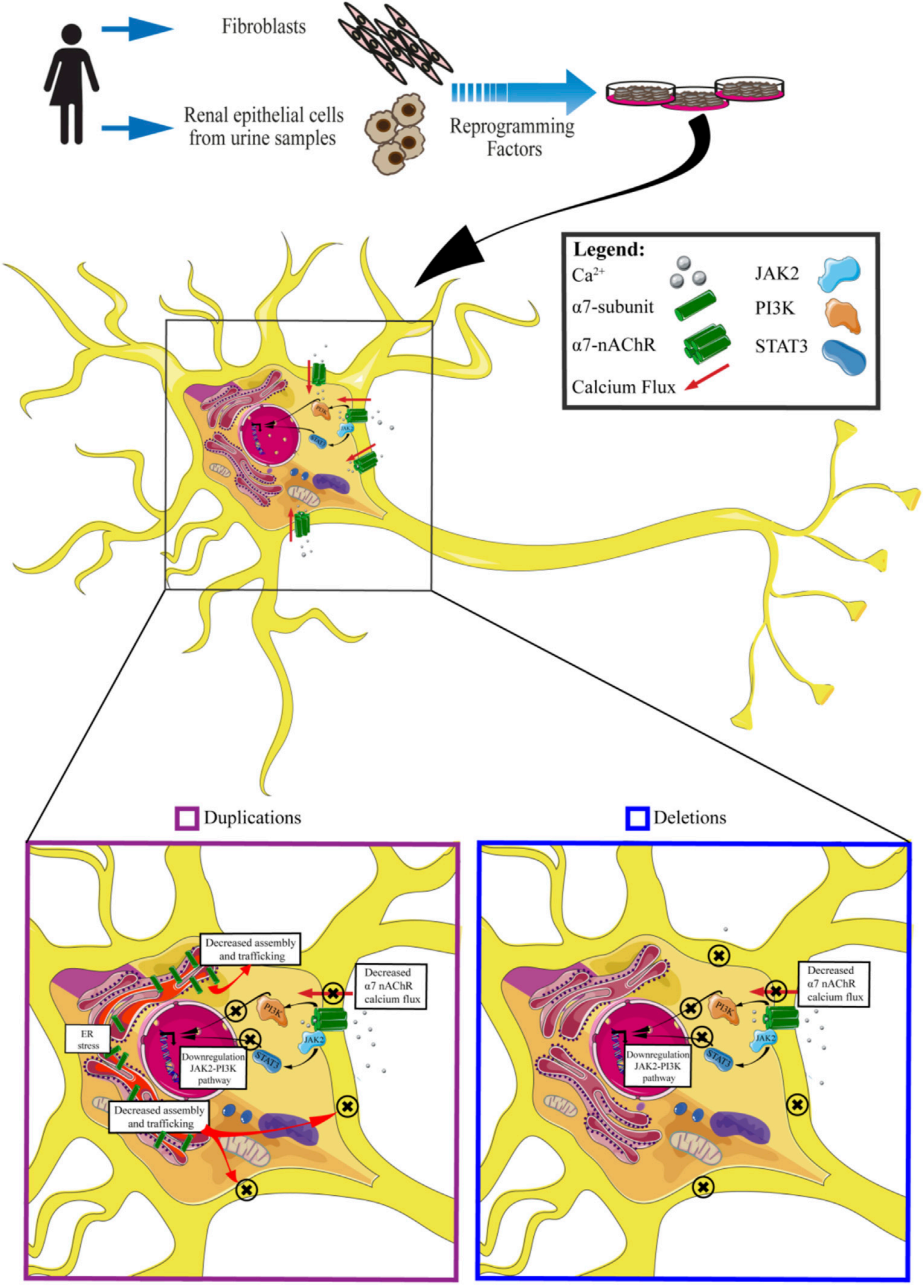
An insight into molecular effects of CNV 15q13.3. Cells carrying CNV duplications show decreased calcium flux associated with the α7 receptor, downregulation of JAK2-PI3K pathway, decreased assembly and trafficking of nAchRs, and ER stress. Cells carrying CNV deletions exhibit decreased α7nAchRs calcium flux and downregulation of JAK2-PI3K pathway.

Thanks to these previous studies it has been possible to highlight many of the molecular mechanisms associated with 15q13.3 CNV: in particular, both in the presence of duplications or deletions, the cellular phenotype is comparable, characterized by a reduction of the calcium flux and its downstream effectors (Figure 4).

### A new perspective on drug development

Neuronal nAChR subtypes, such as a7, have been identified as promising targets for drug development in a variety of neurological and psychiatric disorders (Gill et al., 2013). Therefore, there has been and is a great interest from both academic laboratories and pharmaceutical companies to develop novel subtype-selective nAChR ligands (Hurst et al., 2013). For this reason, the identification of innovative cellular assays, those providing access to native human nAChRs, is an important discovery goal. The research carried out up to now has concentrated mainly on the utilization of receptor agonists and antagonists to explore the physiological behavior of α7 nAChR receptors in the presence and absence of the CNVs, using calcium flux as a readout of receptor functionality. As shown in the preceding paragraphs, the data are very interesting, but an accurate review of all of these published articles points to evidence that the perspective on developing drugs for treating these types of CNV needs to be revised in order to be able to address the mechanisms that influence α7 nAChR both upstream and downstream. Intervening upstream might mean modifying the transport of the receptor towards the membrane, reducing reticular stress, or also reducing the formation of heterodimers between *CHRNA7* e *CHRFAM7A* subunits, while intervening downstream could involve modifying gene expression or epigenetic regulation that has been influenced by dysregulated calcium flux.

## Conclusions

Thanks to the use of hiPSCs it will be possible to study and clarify the role of *CHRNA7* and the contribution of the human fusion gene (*CHRFAM7A*) in neurological-neuropsychiatric diseases. Without the advent of iPSCs technology, these types of studies would have been unthinkable, due to the impossibility of collecting neural tissue from living patients. In this specific context, hiPSC have been an enormous benefit, not only for their capacity of differentiation or the maintenance of the patients’ genetic background but, above all, for the possibility of using pharmacological drugs, to understand both the functionality of the receptors and their response to drugs in physiological conditions, and in the presence of CNVs, to improve pathological functions (Table 1).

**TABLE 1:** Summary of the studies based on the hiPSCs model for studying 15q13.3 CNV.

Authors	Cell type of origin	Type of mutation	Gene expression analysis	Calcium assays	Pharmacological characterization	ER stress	Aβ_1-42_ uptake	Interneuron migration	DNA analysis
Gill et al., 2013	Fibroblasts	—	—	Whole-cell patch-clamp recordings, fluorescence-based calcium imaging	With TQS, 4BP-TQs, and MLA	—	—	—	—
Chatzidaki et al., 2015	Fibroblasts	—	CHRNA7 and CHRFAM7A	FLIPR-based assay	With Type II PAM (PNU-120596) and MLA	—	—	—	—
				Calcium imaging, Patchclamp recording					
Gillentine et al., 2017	Fibroblasts	CHRNA7 deletions and duplications	CHRNA7 (higher in duplications and lower in deletions)	FLIPR-based assay	With Type II PAM (PNU-120596) and MLA	Increased in duplicated lines	—	—	—
Turco et al., 2018	Fibroblasts	Single gene duplication (CHRNA7)	—	—	—	—	—	—	—
Larsen et al., 2019	Fibroblasts	Yes, but not available	CHRNA7 and CHRFAM7A	Calcium imaging	With Type-II PAM (PNU-120596) and Type-I/II (JNJ-39393406, AF58801)	—	—	—	—
Ihnatovych et al., 2019	Fibroblasts	CHRFAM7A null, CHRFAM7A 1 copy	CHRNA7 and CHRFAM7A (which increases during differentiation in 1-copy line)	Single cell-attached and whole-cell patch-clamp recording (reduced activity in 1-copy line)	With Type-II PAM (PNU 120596) (faster desensitization in 1-copy line)	—	Fluorescence imaging and ELISA assay (decreased in 1-copy line)	—	—
Szigeti et al., 2020	Fibroblasts	CHRFAM7A null, CHRFAM7A 1 copy, Transfected CHRFAM7A	CHRFAM7A	Single cell-attached and whole-cell patch-clamp recording	—	—	Fluorescence imaging and ELISA assay (decreased in 1-copy and transfected lines)	—	—
Ihnatovych et al., 2020	Fibroblasts	CHRFAM7A null, CHRFAM7A 1 copy Transfected CHRFAM7A	CHRNA7 and CHRFAM7A	—	—	—	Fluorescence imaging and ELISA assay(decreased in 1-copy and transfected lines)	—	—
Meganathan et al., 2021	Renal epithelial cells	Single gene duplication (CHRNA7)	CHRNA7 (increased in duplicated lines)	Whole-cell voltage and current-clamp recording (increased choline responsiveness and decreased Ach one in duplicated lines)	—	Increased in the affected proband	—	Organoid-based neuronal migration assay (diminished in the affected proband)	—
Zhang et al., 2021	Fibroblasts	CHRNA7 deletions	—	—	—	—	—	—	Methyl-Seq and ATAC-Seq analysis

However, the phenotypic complexity and the variable penetrance associated with CNVs requires the definition of common guidelines to be followed by all researchers working with patients’ iPSC-derived cells so that the results, obtained in different laboratories, can be compared and above all, the information obtained from different experiments can be put together and analyzed globally. The findings so far published are extremely interesting, having highlighted the complexity of receptor functioning, but as can be seen in Figure 3, comparing the CNVs studied in the different articles, it is evident that it is impossible to have a complete and unified picture of the findings. When studies are carried out on deleted CNV, it is important to take in account if the truncation includes also other genes, because either the deregulation of their expression or function could influence the pathological phenotype, as some published articles have highlighted. Likewise, in the presence of duplication, it is necessary to verify the localization of it (where it starts and stops), whether it completely duplicates the gene or whether it is positioned casually inside the gene, provoking a fused protein that may be deregulated in the functionality. Putting all this information together would help researchers unify the data. Moreover, it is essential to consider the specific genetic background of the affected subjects, as well as the specific profile of healthy controls, evaluating the absence of any kind of alteration and defining the homo/heterozygosity of the *CHRFAM7A* locus and the presence or absence of CHRFAM7Abp*.* This is fundamental because studies carried out on the CHRNA7 receptor without considering the presence of *CHRFAM7A* would be incomplete, and potentially lead to erroneous conclusions. Without this information, subsequent evaluations of drug effects could encounter important criticalities because the results are influenced by the genetic background of the patient. Thus, using models with a specific genetic background is important not only for the molecular and functional study of the receptor but also for the transferability of the results into clinical practice, developing drugs that can act in accordance with the specific patients’ genetic profiles. For this purpose, genome editing will accelerate the understanding of the causal relationship between CNV and disease phenotype, as creating the same CNV in different genetic backgrounds, obviously characterized by the presence/absence of the main modifiers of the nicotinic receptor function, the contribution of additional risk alleles to a cell phenotype will be evident.
