# Corrigendum: Deepening the understanding of CNVs on chromosome 15q11–13 by using hiPSCs: An overview

**DOI:** 10.3389/fcell.2023.1141334

**Published:** 2023-02-01

**Authors:** Angela Maria Giada Giovenale, Giorgia Ruotolo, Amata Amy Soriano, Elisa Maria Turco, Giovannina Rotundo, Alessia Casamassa, Angela D’Anzi, Angelo Luigi Vescovi, Jessica Rosati

**Affiliations:** ^1^ Cellular Reprogramming Unit, Fondazione IRCCS Casa Sollievo della Sofferenza, San GiovanniRotondo, Italy; ^2^ Department of Biotechnology and Biosciences, University of Milano-Bicocca, Milan, Italy

**Keywords:** neurodevelopmental disorders, neuropsychiatric disorders, 15q11-13, CHRNA7, nicotinic acetylcholine receptor, copy number variation, CNV

In the published article, there is an error in Figure 3 and Figure 4 as published. The two figures are inverted, while the captions are correct**.** The corrected Figure 3 and Figure 4 and its caption appear below.

**FIGURE 3:**
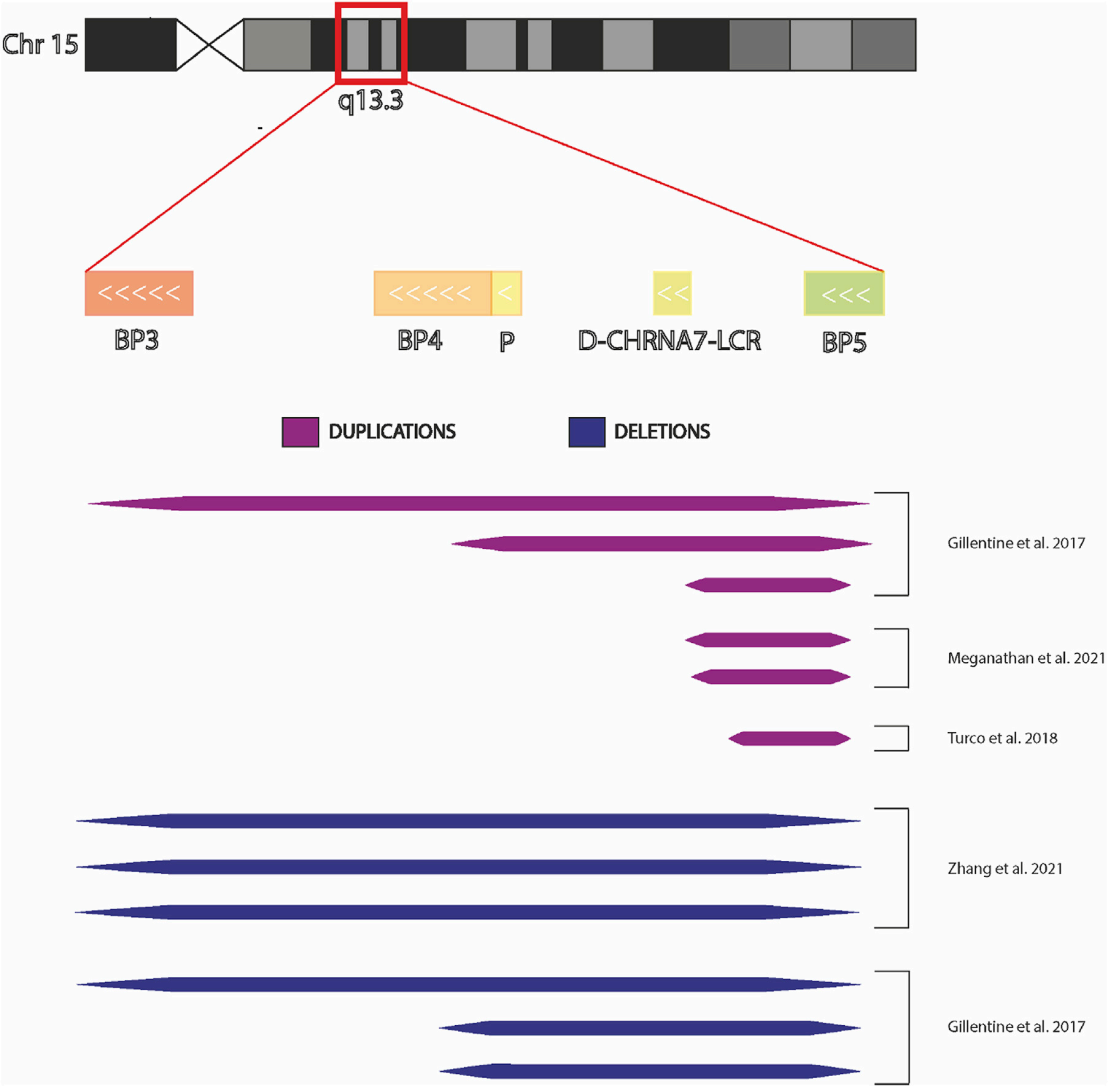
Chromosome 15q13.3 and hiPSCs derived from individuals with CNV. Graphic representation of chromosomal region 15q13.3 showing BreakPoint regions BP3, BP4 and BP5 and the extensions the microdeletions and microduplications present in the hiPSCs in the published studies.

**FIGURE 4:**
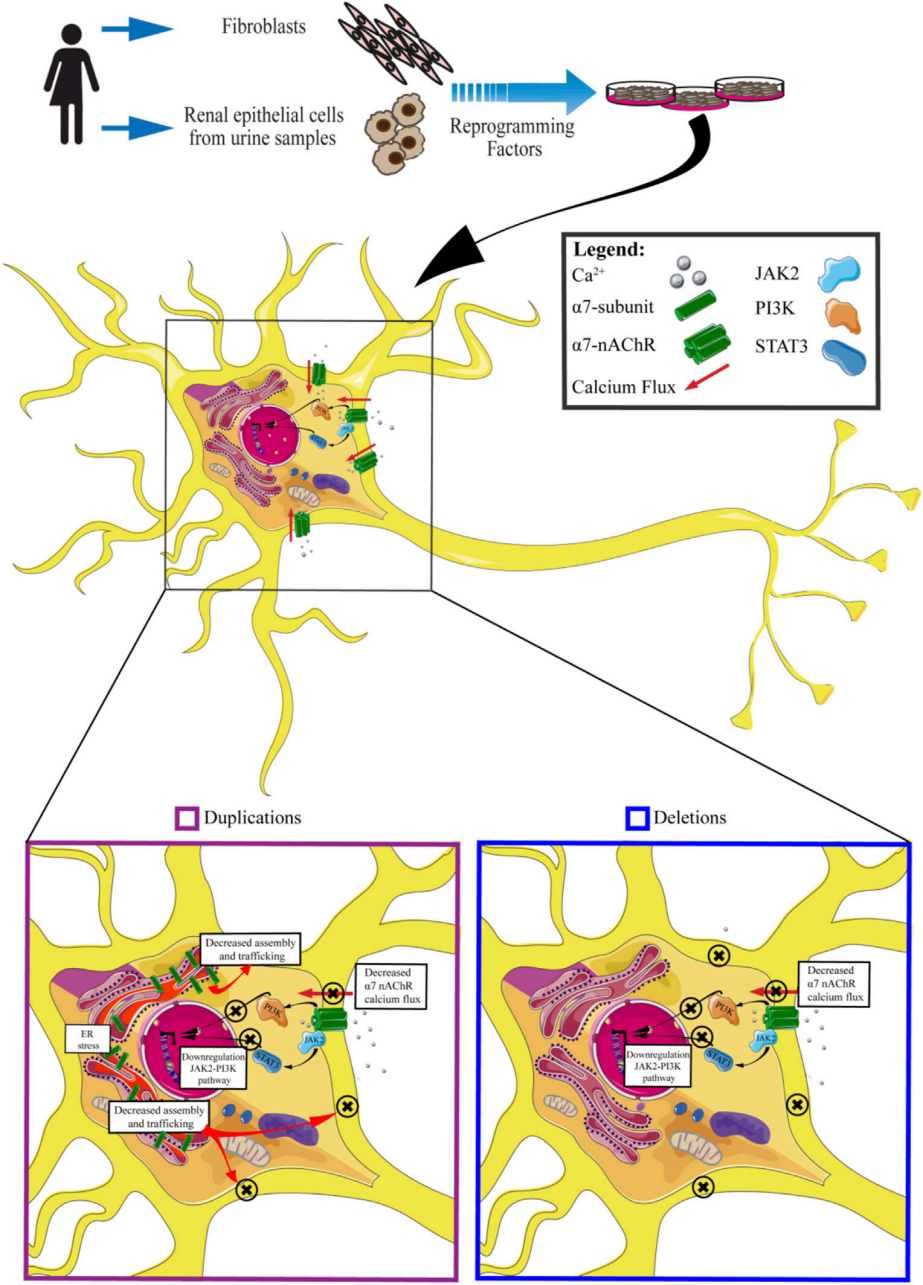
An insight into molecular effects of CNV 15q13.3. Cells carrying CNV duplications show decreased calcium flux associated with the α7 receptor, downregulation of JAK2-PI3K pathway, decreased assembly and trafficking of nAchRs, and ER stress. Cells carrying CNV deletions exhibit decreased α7nAchRs calcium flux and downregulation of JAK2-PI3K pathway.

In the published article, there is an error in Table 1 as published. The table is not paginated correctly**.** Table 1 and its caption appear below.

**TABLE 1:** Summary of the studies based on the hiPSCs model for studying 15q13.3 CNV.

Authors	Cell type of origin	Type of mutation	Gene expression analysis	Calcium assays	Pharmacological characterization	ER stress	Aβ_1-42_ uptake	Interneuron migration	DNA analysis
Gill et al., 2013	Fibroblasts	—	—	Whole-cell patch-clamp recordings, fluorescence-based calcium imaging	With TQS, 4BP-TQs, and MLA	—	—	—	—
Chatzidaki et al., 2015	Fibroblasts	—	CHRNA7 and CHRFAM7A	FLIPR-based assay	With Type II PAM (PNU-120596) and MLA	—	—	—	—
				Calcium imaging, Patchclamp recording					
Gillentine et al., 2017	Fibroblasts	CHRNA7 deletions and duplications	CHRNA7 (higher in duplications and lower in deletions)	FLIPR-based assay	With Type II PAM (PNU-120596) and MLA	Increased in duplicated lines	—	—	—
Turco et al., 2018	Fibroblasts	Single gene duplication (CHRNA7)	—	—	—	—	—	—	—
Larsen et al., 2019	Fibroblasts	Yes, but not available	CHRNA7 and CHRFAM7A	Calcium imaging	With Type-II PAM (PNU-120596) and Type-I/II (JNJ-39393406, AF58801)	—	—	—	—
Ihnatovych et al., 2019	Fibroblasts	CHRFAM7A null, CHRFAM7A 1 copy	CHRNA7 and CHRFAM7A (which increases during differentiation in 1-copy line)	Single cell-attached and whole-cell patch-clamp recording (reduced activity in 1-copy line)	With Type-II PAM (PNU 120596) (faster desensitization in 1-copy line)	—	Fluorescence imaging and ELISA assay (decreased in 1-copy line)	—	—
Szigeti et al., 2020	Fibroblasts	CHRFAM7A null, CHRFAM7A 1 copy, Transfected CHRFAM7A	CHRFAM7A	Single cell-attached and whole-cell patch-clamp recording	—	—	Fluorescence imaging and ELISA assay (decreased in 1-copy and transfected lines)	—	—
Ihnatovych et al., 2020	Fibroblasts	CHRFAM7A null, CHRFAM7A 1 copy Transfected CHRFAM7A	CHRNA7 and CHRFAM7A	—	—	—	Fluorescence imaging and ELISA assay(decreased in 1-copy and transfected lines)	—	—
Meganathan et al., 2021	Renal epithelial cells	Single gene duplication (CHRNA7)	CHRNA7 (increased in duplicated lines)	Whole-cell voltage and current-clamp recording (increased choline responsiveness and decreased Ach one in duplicated lines)	—	Increased in the affected proband	—	Organoid-based neuronal migration assay (diminished in the affected proband)	—
Zhang et al., 2021	Fibroblasts	CHRNA7 deletions	—	—	—	—	—	—	Methyl-Seq and ATAC-Seq analysis

The authors apologize for this error and state that this does not change the scientific conclusions of the article in any way. The original article has been updated.

